# Gelatin methacryloyl (GelMA) loaded with concentrated hypoxic pretreated adipose-derived mesenchymal stem cells(ADSCs) conditioned medium promotes wound healing and vascular regeneration in aged skin

**DOI:** 10.1186/s40824-023-00352-3

**Published:** 2023-02-13

**Authors:** Shiyi Li, Jiachen Sun, Jinxiu Yang, Yi Yang, Hongfan Ding, Boya Yu, Kui Ma, Minliang Chen

**Affiliations:** 1 Senior Department of Burns and Plastic Surgery, The Fourth Medical Center of Chinese PLA General Hospital, No. 51 Fucheng Road, Haidian District, Beijing, 100038 China; 2 Chinese PLA Medical School, Beijing, 100853 China; 3 Department of Dermatology and Venerology, Peking University First Hospital, Beijing, 100034 China; 4 7th Department of Plastic Surgery, Plastic Surgery Hospital, Chinese Academy of Medical Sciences and Peking Union Medical College, Beijing, 100144 China; 5 Research Center for Tissue Repair and Regeneration Affiliated to the Medical Innovation Research Division, Chinese PLA General Hospital, Beijing, 100048 China

**Keywords:** Aged wound, Angiogenesis, GelMA hydrogel, ADSCs, Conditioned medium, Hypoxia

## Abstract

**Background:**

Aging skin is characterized by a disturbed structure and lack of blood supply, which makes it difficult to heal once injured. ADSCs secrete large amounts of cytokines, which promote wound healing and vascular regeneration through paracrine secretion, and the number of cytokines can be elevated by hypoxic pretreating. However, the components of ADSCs are difficult to retain in wounds. Gelatin methacrylate (GelMA) is a photopolymerizable hydrogel synthesized from gelatin and has recently emerged as a potentially attractive material for tissue engineering applications. GelMA loaded with concentrated hypoxic pretreated ADSCs conditioned medium could provide a new method of treating wounds in aged skin.

**Methods:**

Primary ADSCs were isolated from human adipose tissue and characterized by flow cytometry and differentiation test. ADSCs in passages 4-6 were pretreated in the hypoxic and normoxic environments to collect conditioned medium, the conditioned medium was then concentrated to prepare concentrated ADSCs conditioned medium(cADSC-CM)(the one collected from ADSCs under hypoxia was called hypo-CM ,and the one from normoxia was called nor-CM). The concentration of cytokines was detected. After treated with cADSC-CM, the abilities of proliferation, migration, and tube formation of human umbilical vascular endothelial cells (HUVECs) were assayed, and Akt/mTOR and MAPK signal pathway was detected using western blotting. GelMA+hypo-CM hydrogel was prepared, and a comprehensive evaluation of morphology, protein release efficiency, degradation rate, mechanical properties, and rheology properties were performed. Full-thickness skin wounds were created on the backs of 20-month-old mice. After surgery, GelMA, GelMA+F12, GelMA+hypo-CM, and GelMA+nor-CM were applied to the wound surface respectively. H&E, Masson, and immunohistochemistry staining were performed, and a laser Doppler perfusion imager was used to evaluate the blood perfusion. The student’s t-test was used for analysis between two groups and a one-way analysis of variance (ANOVA) was used for analysis among multi groups.

**Results:**

Our results revealed that 1) wounds in aged skin healed more slowly than that in young skin and exhibited poorer perfusion; 2) hypoxic pretreated ADSCs secreted more cytokines including VEGF by activating HIF1α; 3) hypo-CM promoted proliferation and migration of HUVECs through VEGF/Akt/mTOR and MAPK signal pathway; 4) GelMA-hypoCM accelerated wound healing and angiogenesis in aged skin in vivo.

**Conclusion:**

GelMA loaded with concentrated hypoxic pretreated adipose-derived mesenchymal stem cells conditioned medium could accelerate wound healing in aged skin by promoting angiogenesis.

**Supplementary Information:**

The online version contains supplementary material available at 10.1186/s40824-023-00352-3.

## Introduction

The skin acts as a key physical barrier to the external environment, preventing the loss of moisture and other body components and protecting the body from potential threats. After an injury, the wound healing response is rapidly activated to repair and restore the skin barrier. Wound healing is a complex biological process involving many cells and cytokines [1]. Poor underlying conditions during re-epithelialization can easily lead to delayed wound healing [2–4], which will undoubtedly cause more pain to the patient.

Neovascularization is an essential component of wound healing since it has a fundamental impact from the beginning after skin injury to the end of wound remodeling [5]. Neovascularization provides sufficient oxygen to the impaired wound where cells at the wound edges and in granulation tissue consume large amounts of oxygen, and fibroblasts require sufficient oxygen to produce the collagen necessary for wound healing [6]. Skin is one of the organs that are prone to aging, as evidenced by dryness, roughness, delayed wound healing, and increased susceptibility to infection. Extensive research has shown that angiogenesis is delayed by aging [4, 7–9]. It also has previously been observed that there was a lack of expression of vascular endothelial growth factor (VEGF) in aged skin wounds compared to younger skin [10, 11].

When surgeons treat chronic wounds, they apply various growth factor drugs in addition to routine disinfection and dressing changes, an approach that often does not yield good results in older patients. Adipose-derived mesenchymal stem cells (ADSCs) have shown prominence in the field of regenerative medicine because they can be easier to obtain and have a wide variety of sources. ADSCs, characterized by the secretion of many cytokines, are currently considered potential therapeutic strategies for several diseases including chronic wounds [12–14]. Recently, considerable evidence has accumulated to show that ADSCs can promote the healing and angiogenesis of chronic wounds through paracrine secretion, which brings hope to the treatment of chronic wounds [13, 15]. What’s more, It is now well established from a variety of studies that hypoxia pretreated ADSCs are more biologically active and secreted more cytokines, which is of great potential for stem cell therapy [16–18]. The mechanism may be that the hypoxic environment allows hypoxia-inducible factor 1 (HIF1) to be continuously activated. HIF-1 consists of two subunits: HIF-1α and HIF-1β, which bind to regulate the expression of a lot of genes related to biological processes. Under hypoxic conditions, HIF-1α expression was higher and sustained, whereas, under normoxic conditions, HIF-1α was rapidly degraded post-translationally [19].

However, most of the previous studies on stem cell components and wound healing have been done by injecting stem cell components directly into the wound edges or underneath the wound. This method is certainly difficult to retain the stem cell component and requires multiple treatments. Therefore, finding an appropriate wound dressing that can be loaded with stem cell components is a hot topic of research. Gelatin methacrylate (GelMA) is a photopolymerizable hydrogel synthesized from gelatin and has recently emerged as a potentially attractive material for tissue engineering applications [20–24]. GelMA has been applied in the field of tissue engineering and regenerative medicine due to its injectable and UV-cross-linked properties. Research in this area has shown that GelMA loaded with various cellular components had excellent results in promoting wound healing [25–27].

**Fig. 1:**
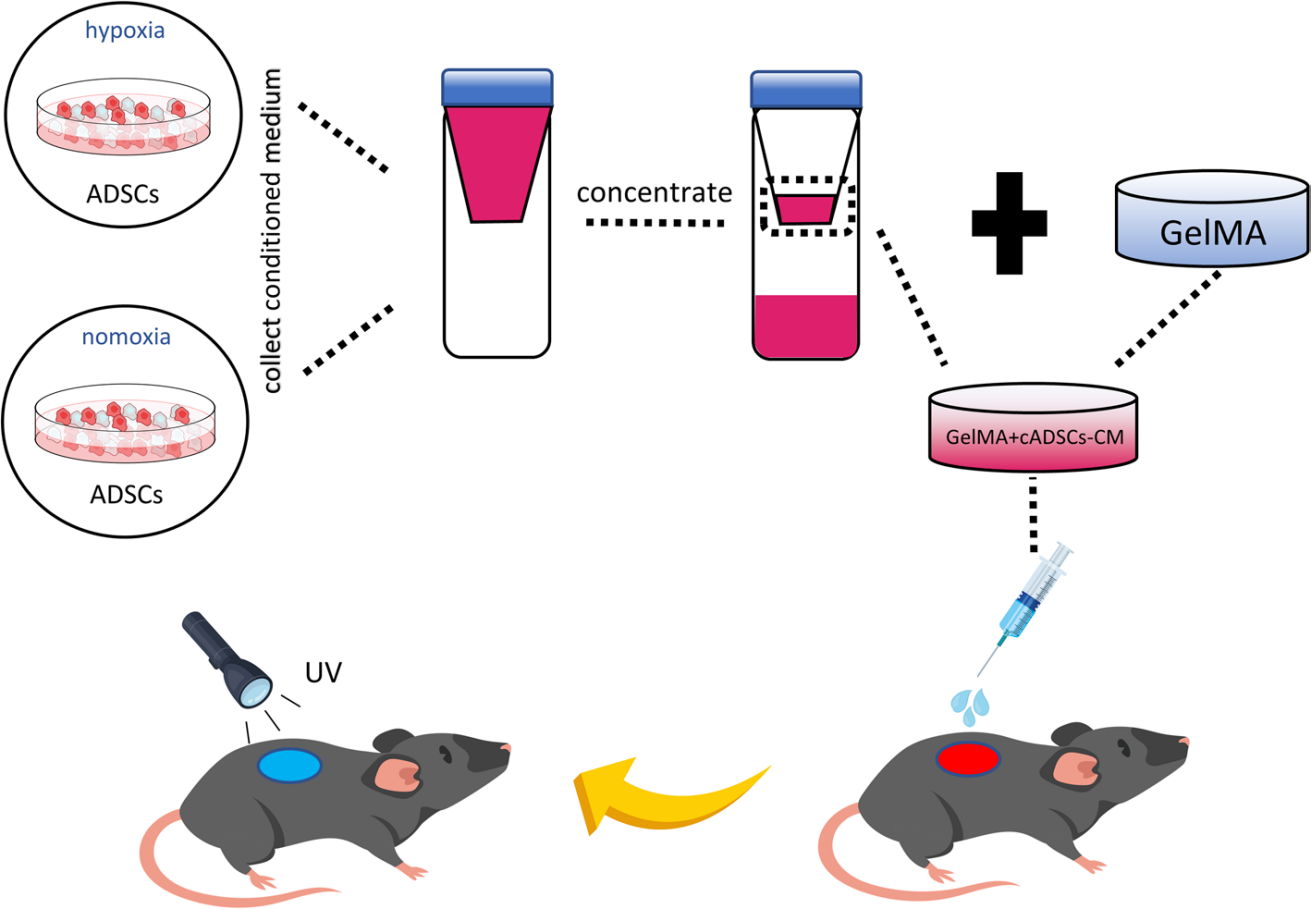
Schematic diagram of the experimental procedure for in vivo studies

In this study, we set out to investigate the effect of GelMA loaded with concentrated hypoxic pretreated ADSCs conditioned medium(cADSCs-CM) on wound healing. ADSCs pretreated with hypoxic and normoxic were used to collect cADSCs-CM and the cytokines in cADSCs-CM were detected. The human umbilical vascular endothelial cells (HUVECs) were treated with cADSCs-CM and the abilities of proliferation, migration, and tube formation of HUVECs were evaluated to observe the angiogenic efficacy of cADSCs-CM. We subsequently mixed GelMA with cADSC-CM to prepare a GelMA + cADSC-CM hydrogel which was applied as a dress for wounds in aged skin(Fig 1). The effects of GelMA + cADSC-CM hydrogel on aged wound healing and angiogenesis were assessed.

## Methods

### Obtain and culture of ADSCs and HUVECs

Human adipose tissue was obtained from the waste fat of patients who had undergone liposuction, and the liposuction site was the thighs on both sides. All enrolled patients signed an informed consent form.

The adipose tissue was rinsed three times with phosphate-buffered saline (PBS, Solarbio, Beijing, China), and then 0.1% type I collagenase (Sigma–Aldrich, St. Louis, MO, USA) was used to digest the adipose tissue for 50 minutes. Then, the stromal vascular fraction was filtered through a 70 μm porous filter (Millipore, USA) after centrifugation at 2000 rpm for 10 minutes. After that, ADSCs were resuspended in Dulbecco's modified Eagle’s medium: F-12 (DMEM/F-12, Gibco BRL, NY, USA) containing 10% fetal bovine serum (FBS, Gibco BRL, NY, USA) and 1% penicillin/streptomycin (Solarbio, Beijing, China) at 37°C in an incubator with 5% carbon dioxide (CO_2_). The medium was changed every two days.

The cell line of HUVECs was purchased from the American Type Culture Collection (ATCC, USA). HUVECs were cultured in Dulbecco's modified Eagle’s medium(DMEM, Gibco BRL, NY, USA) containing 10% FBS and 1% penicillin/streptomycin at 37°C in an incubator with 5% CO_2._ The medium was changed every two days.

### Flow Cytometry to identify ADSCs

Flow cytometry was used to identify the phenotype of the cultured ADSCs. The mesenchymal stem cell surface marker detection kit (OriCell Bioscience, Inc., China, HUXMX-09011) was used in this test. Briefly, according to the manufacturer’s instruction, ADSCs were collected and then incubated with purified antibodies against CD90, CD34, CD105, CD31, CD45, and CD73 at 4 °C for 30 min in the dark. After washing twice, the cells were incubated with phycoerythrin (PE) goat anti-mouse IgG antibody and detected by a FACSCalibur instrument (BD Biosciences, CA, USA). Data were analyzed using Flowjo software(TreeStar, Inc., Ashland, OR, USA).

### Adipogenic, osteogenic and chondrogenic differentiation

ADSCs were inoculated in six-well plates (Corning, NY, USA) at a cell density of 2×10^5^/well until the confluence of cells reached 80%. Adipogenic differentiation was proceeded using basic medium A containing 10% FBS, 1% penicillin-streptomycin, 1% glutamine, 0.2% insulin, 0.1% 3-isobutyl-1-methyl xanthine, 0.1% rosiglitazone, and 0.1% dexamethasone for 3 days and basic medium B containing 10% FBS, 1% penicillin-streptomycin, 1% glutamine, and 0.2% insulin for 1 day, and they alternated 4 times (Cyagen Bioscience, Inc., China, HUXMD-90031).

Osteogenic differentiation was proceeded using basic medium containing 10% FBS, 1% penicillin-streptomycin, 1% glutamine, 0.2% ascorbate, 1% β-glycerophosphate, and 0.01% dexamethasone for 3 weeks (Cyagen Bioscience, Inc., China, HUXMD-90021).

At the end of induction, 4% paraformaldehyde (Solarbio, Beijing, China) was used to immobilize the cells for 30 min, and Oil Red O and Alizarin Red S dye solutions were used to assess adipogenic and osteogenic differentiation according to the manufacturer’s instruction respectively. After staining, the cells were observed under a microscope (Olympus, Tokyo, Japan).

ADSCs were harvested and resuspended in a centrifuge tube at a cell density of 4×10^5^/tube. The medium contained 0.3% ascorbate, 0.01% dexamethasone, 1% insulin ferro-selenium transporter supplement, 0.1% sodium pyruvate, 0.1% proline and 1% transforming growth factor-β3 (Cyagen Bioscience, Inc., China, HUXMD-90041). The cells were cultured at 37 °C in 5% CO_2_ for 21 days. After induction, 4% paraformaldehyde was used to immobilize the cartilage balls for 30 min at room temperature, and Alcian blue staining was used to assess chondrogenic differentiation according to the manufacturer’s instruction.

### Preparation of cADSCs-CM

ADSCs in passages 4-6 were used to collect conditioned medium. For the hypoxic group, ADSCs that were 80 - 90% confluent were starved with serum-free DMEM/F12 medium at 37°C in an incubator with 1% oxygen and 5% CO_2_ for 48h. For the normoxic group, ADSCs were incubated in an incubator with 5% CO_2._ Then, the ADSCs supernatant was collected, centrifuged at 3500 rpm for 10 minutes, and filtered using a 0.22 μm Millex GP syringe filter (Millipore, USA) to ensure sterility. The product is ADSC-CM. After that, ADSC-CM was transferred to an ultrafiltration centrifugal tube(Millipore, USA, UFC9003) and centrifuged at 3500 rpm for 25 minutes. The upper product used as cADSCs-CM was collected and stored at -80°C until use. For convenience, in this study, the cADSC-CM obtained in the hypoxic environment was called hypo-CM, and the one obtained in the normoxic environment was called nor-CM.

### Luminex liquid suspension chip detection

The Bio-Plex Pro Human Cytokine Screening 48-plex panel was used following the manufacturer's instructions. In brief, The hypo-CM and nor-CM processed according to the instructions were incubated in a 96-well plate embedded with microbeads for 30 min and then incubated with detection antibody for 30 min. Subsequently, Streptavidin-PE was added to each well, and fluorescence values were calculated using BioPlex System(Bio-Rad, Hercules, CA, USA).

### CCK-8 assay

Cell proliferation was determined by using Cell Counting Kit-8 (Beyotime Biotech-nology, Shanghai, China). HUVECs (3000 / well) were treated with different mediums (F12; hypo-CM; hypo-CM+DMSO; hypo-CM+ cabozantinib; nor-CM; nor-CM+DMSO; nor-CM+ cabozantinib) for 48 h in 96-well plates. The optical density at 450 nm was measured on a multiwell plate reader(Tecan, USA) every 24 h.

### Immunofluorescence

For immunolabeling, HUVECs treated with different mediums(F12; hypo-CM; hypo-CM+DMSO; hypo-CM+ cabozantinib; nor-CM; nor-CM+DMSO; nor-CM+ cabozantinib) were fixed in 4% paraformaldehyde for 15 min and permeabilized in PBS with 0.1% Triton X-100(HFH10, Invitrogen) for 10 min at room temperature. Nonspecific binding sites were blocked for 1 h by PBS containing 1% bovine serum albumin and 0.1% Tween 20. The fixed cells were incubated overnight at 4 °C with antibodies specific for the Ki67 mouse monoclonal antibody (9449, 1:500, CST), Specific labeling was visualized using secondary antibodies conjugated with Alexa 647 (ab150115, 1:200, Abcam). Nuclei were visualized by staining with DAPI (Thermo Fisher, Waltham, MA, USA). Images were acquired with a confocal microscope (SP8, Leica).

### Cells Migration assays

The migration property was evaluated by scratch assay. HUVECs were cultured in a six-well plate, and when 80% confluence was reached, the cells were scratched with a

pipet tip through the well bottom center and treated with different mediums(as above described). Images were taken using a microscope (Olympus, Tokyo, Japan) every 12 h. ImageJ software was used to measure the area and length of the scratches to calculate the average width of the scratches. The migration rate of the scratch was calculated as follows: migration rate (%) = (W0 − Wt)/W0 × 100%, where W0 is the original width and Wt is the remaining width at the measured time point.

### Tube formation assay

HUVECs(2×10^4^/well) were seeded into Matrigel-coated 96-well plates and then treated with different mediums as those in immunofluorescence assay for 10 h. The tube formation images were acquired using a microscope, and the branch length was calculated using ImageJ software.

### Western blot

To figure out the signaling pathway of ADSCs in hypoxic environments, BAY87-2243(5 μM, S7309, Selleck Chemicals, USA) was used to block HIF-1. ADSCs were treated with different mediums (F12, F12+DMSO, F12+ BAY87-2243) and cultured in the hypoxic environment or normoxic environment, respectively. HUVECs were treated as above described.

Radioimmunoprecipitation assay buffer (Beyotime, China) was used to extract proteins from ADSCs and HUVECs. Samples (60 μg protein) were separated on 10% SDS–PAGE gels and transferred to a polyvinylidene fluoride membrane, blocked with 5% nonfat dried milk in TBST (10 mmol/L Tris, pH 7.5; 150 mmol/L NaCl, 0.05% Tween-20), incubated with primary antibodies including HIF-1α mouse monoclonal antibody(66730-1-Ig, 1:2000, Proteintech, Wuhan, China), VEGFA mouse monoclonal antibody(66828-1-Ig, 1:1000, Proteintech, Wuhan, China), β-actin rabbit monoclonal antibody(3700, 1:1000, CST, USA), mTOR rabbit monoclonal antibody(2983, 1:1000, CST, USA), p-mTOR rabbit monoclonal antibody(5536, 1:1000, CST, USA), Akt rabbit monoclonal antibody (4691, 1:1000, CST, USA), p-Akt rabbit monoclonal antibody (4060, 1:1000, CST, USA), MEK rabbit polyclonal antibody (11049-1-AP, 1:1000, Proteintech, Wuhan, China), p-MEK rabbit monoclonal antibody (9154, 1:1000, CST, USA), Erk rabbit polyclonal antibody (11257-1-AP, 1:1000, Proteintech, Wuhan, China), p-Erk rabbit monoclonal antibody (4370, 1:1000, CST, USA) and gapdh mouse monoclonal antibody (60004-1-Ig, 1:50000, Proteintech, Wuhan, China) at 4 °C overnight. After washing with TBST, the membranes were incubated with horseradish peroxidase-conjugated goat anti-rabbit or anti-mouse secondary antibodies (EpiZyme Biotech, Shanghai, China). The immunoreactive bands were developed using an ECL kit (Thermo Fisher Scientific, Waltham, MA, USA) and detected with the Bio-Rad Molecular Imager Gel Doc TM XR+ (Bio-Rad, Hercules, CA, USA). Protein expression levels were quantified by densitometry analysis using Image J software (NIH, USA).

### Preparation of GelMA-hypoCM hydrogels and analysis of proteins release

GelMA and lithiumphenyl-2,4,6-trimethylbenzoyl phosphinate (LAP) photoinitiator were purchased from Engineering for Life Co., Ltd (Suzhou, China). LAP photoinitiator standard solution with a concentration of 0.25% was prepared according to the instruction which was used to prepare 5%, 10%, and 15% GelMA solution. The hydrogel solution was passed through a 0.22 μm filter to ensure sterility. GelMA-hypoCM hydrogels were made with GelMA solution and hypoCM in a 1:1 ratio(v:v) which was chemically cross-linked by blue light (405nm) for 15 s. After lyophilization and gold spraying, the GelMA-hypoCM hydrogel samples were observed and photographed under a scanning electron microscope (ZEISS Gemini 300, Carl Zeiss, Germany). The hydrogels were immersed in PBS in a 24-well plate and the supernatant was collected every day for 15 days. The protein content in the supernatant was detected using BCA Protein Assay Kit(Solarbio, Beijing, China).

### Degradation assay

The cross-linked GelMA-hypoCM hydrogel samples were immersed in PBS at 37°C. After being dried under vacuum at 50°C to remove water, the samples were weighed at predetermined time points. The degradation ratios were calculated as follows: degradation ratio = the hydrogels’ current weight/initial weight × 100%.

### Analysis of mechanical properties and viscosity

The tensile assay was performed using a universal mechanical testing machine(MTS, USA). The cross-linked GelMA-hypoCM hydrogel samples were made into cylinders with a length of 30 mm and a width of 10 mm and horizontally stretched at a speed of 2 mm/min. The stress-strain curve was recorded until the sample was broken.

Rheological properties were tested by a rheometer(Anton Paar, Germany). GelMA-hypoCM hydrogel samples were examined for viscosity change over time at a shear rate of 10 / s and the temperature was constant at 25°C. The viscosity value was recorded every 20 s for 20 min. In addition, cross-linked 10% GelMA-hypoCM hydrogels were used to examine the variation of modulus with temperature for a fixed strain and frequency condition. The temperature was ramped up from 4 to 40 °C and then cooled down from 40 to 4 °C. The storage modulus(G’) and loss modulus(G’’) are measured and recorded.

### Creation of skin defect model in mice and treatment with GelMA-hypoCM

Male C57BL/6J mice (2-month-old and 20-month-old) were purchased from the SPF Biotechnology Co., Ltd. (Beijing, China), and raised in the animal facility of the Fourth Medical Center, Chinese PLA General Hospital following institutional guidelines for one week before the experiment.

The mice were anesthetized with iodophor and full-thickness skin wounds with a diameter of 10 mm were created on their backs. In the first animal experiment, mice were grouped according to age, and wounds in both groups were photographed on day 0, 6, and 12. In the second experiment, only 20-month-old mice were used and they were randomly divided into five groups: a) blank; b) GelMA; c) GelMA+F12 group; d) GelMA+hypo-CM; e) GelMA+nor-CM, each of them with 6 mice. The hydrogels used for treatment were all made of 10% GelMA and the mixing ratio of GelMA with F12, hypo-CM, or nor-CM were 1:1(v:v). Wounds in each group were photographed on day 0, 4, 8, and 12. The mice were euthanized after being anesthetized, and the healing wound tissues were collected for future use.

### Blood perfusion evaluation

Blood perfusion of the healing wounds on day 12 after making wounds was evaluated by a laser Doppler perfusion imager(PeriCam PSI-ZR, PERIMED Ltd, Sweden). In laser Doppler imaging, a collimated laser beam is used to scan the tissue, and 2-dimensional, color-coded blood flow images were captured.

### Histological analysis immunohistochemistry staining

Tissue samples that were collected on day 12 after making wounds of all groups were excised and fixed in 4% paraformaldehyde, embedded in paraffin, sectioned at 5-μm thickness, mounted on slides, and stained with H&E following the instructions. The Masson staining was conducted using the ready-to-use kit (Trichrome Stain (Masson) Kit, HT15, Sigma-Aldrich). Briefly, the tissue was cut into 5μm sections. Sections were immersed in Bouin’s solution (HT 10132, Sigma-Aldrich), stained in Weigert’s hematoxylin, incubated in phosphotungstic-phosphomolybdic acid, dyed with aniline blue, and fixed in 1% acetic acid. Then, the slides were rinsed in distilled water, dehydrated, observed, and photographed under a microscope (Olympus, Tokyo, Japan).

Immunohistochemistry staining for CD31 was performed using an anti-CD31 antibody(1:100, Abcam, USA). Briefly, After dewaxing, hydration, and antigen repair, the sections were incubated with anti-CD31 antibody at 4°C overnight, and then with the secondary HRP-conjugated antibody (1:200, Abcam) for 1 h at room temperature. Diaminobenzidine (DAB) reagent (Beyotime Biotechnology, China) was applied for 3 minutes and hematoxylin was applied for 2 minutes to restain the nuclei. Then, the slides were observed and photographed under a microscope. Average epidermis thickness was calculated using ImageJ software.

### Statistical analysis

The results are presented as mean ± standard deviation (SD). The data were analyzed using SPSS software 25.0(IBM, USA). The student’s t-test was used for analysis between two groups and a one-way analysis of variance (ANOVA) was used for analysis among multi groups. Differences with a P-value of < 0.05 were considered statistically significant(*P<0.05, **P<0.01, ***P<0.001).

## Results

### Wounds in aged skin healed more slowly than that in young skin and exhibit poorer perfusion

**Fig. 2:**
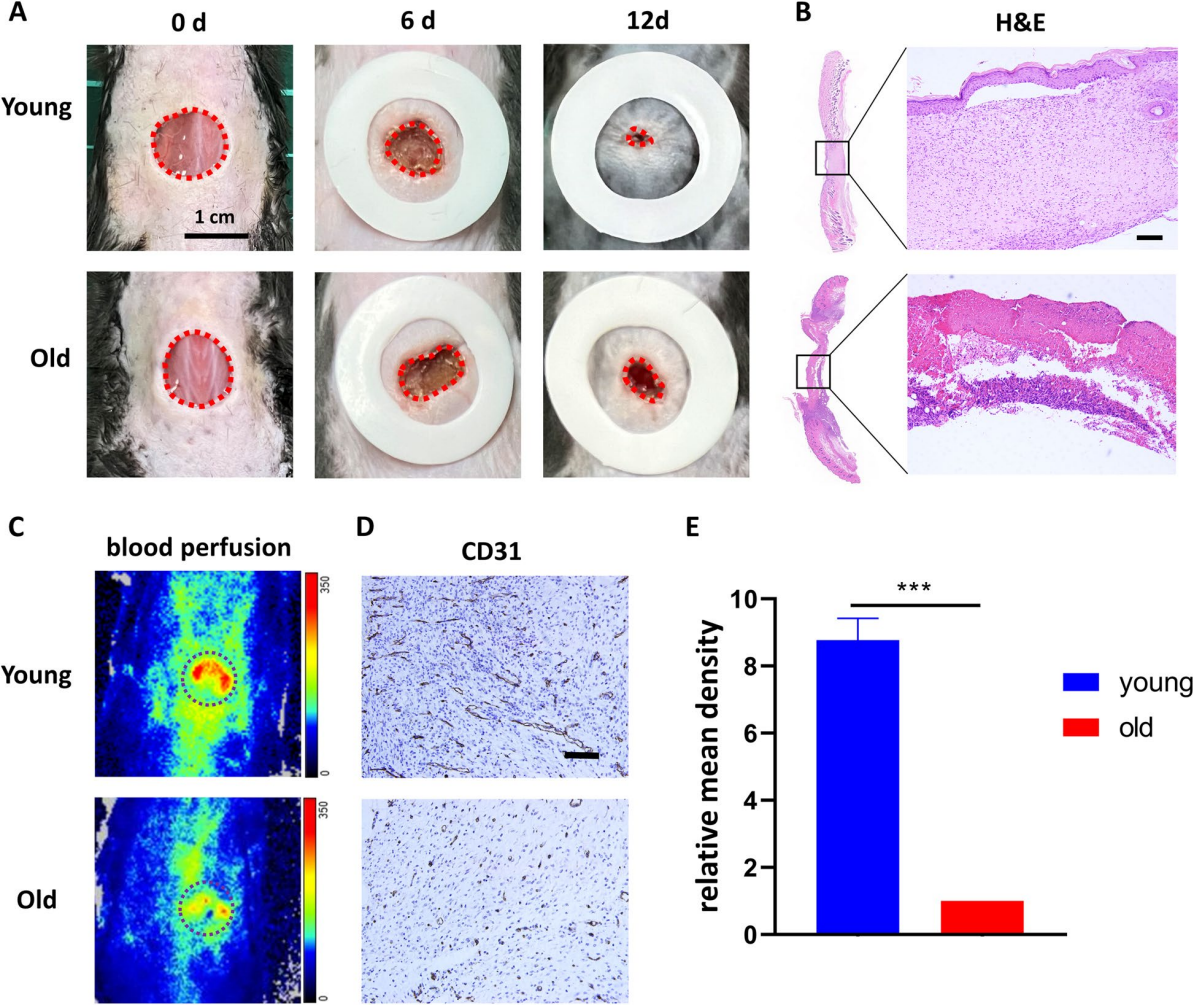
Wounds in aged skin healed more slowly than that in young skin and exhibit poorer perfusion. **A**. The image of wounds closure of young and old groups at day 0, 6, 12. Scale bar = 1 cm. **B**. H&E staining of wound site of young and old groups at day 12. Scale bar = 100 μm. **C**. The blood perfusion at the wound areas of both groups were evaluated by a laser Doppler perfusion imaging system. **D**. Immunohistochemical staining of CD31 in regenerated skin tissue. Scale bar = 50 μm. **E**. Quantitative analysis of relative mean density of immunohistochemical staining of CD31 in regenerated skin tissue. The results are presented as mean ± SD (***P<0.001).

To compare the healing rate of young and old wounds, we used six 2-month-old and six 20-month-old C57BL/6J mice, respectively, and created 10-mm-diameter full skin defects on their backs. The area of the wounds was recorded on days 0, 6, and 12, and the observation of wounds at different time points was displayed in Fig. 2A. It is apparent from Fig. 2A that the wound was almost completely healed by day 12 in younger skin, however, older skin still has most of the skin defects. Histopathological staining images demonstrated the healing effect more visually. As illustrated in Fig. 2B, on day 12, the young skin already has a continuous epidermal layer, at the same time, a large number of fibroblasts and collagen deposits in the dermis can be seen. However, in wounds of older skin, no continuous skin tissue can be found under the scabs.

It is undeniable for every clinician that blood perfusion is essential for wound healing, so we applied a laser Doppler perfusion imager to examine blood perfusion at the trauma site and immunohistochemical staining of CD31 to verify microangiogenesis. Not surprisingly, as shown in Fig. 2C and 2D, the wound site of older skin has lower blood perfusion and a very low number of microvessels compared to that in young skin.

### Characterization of ADSCs

**Fig. 3:**
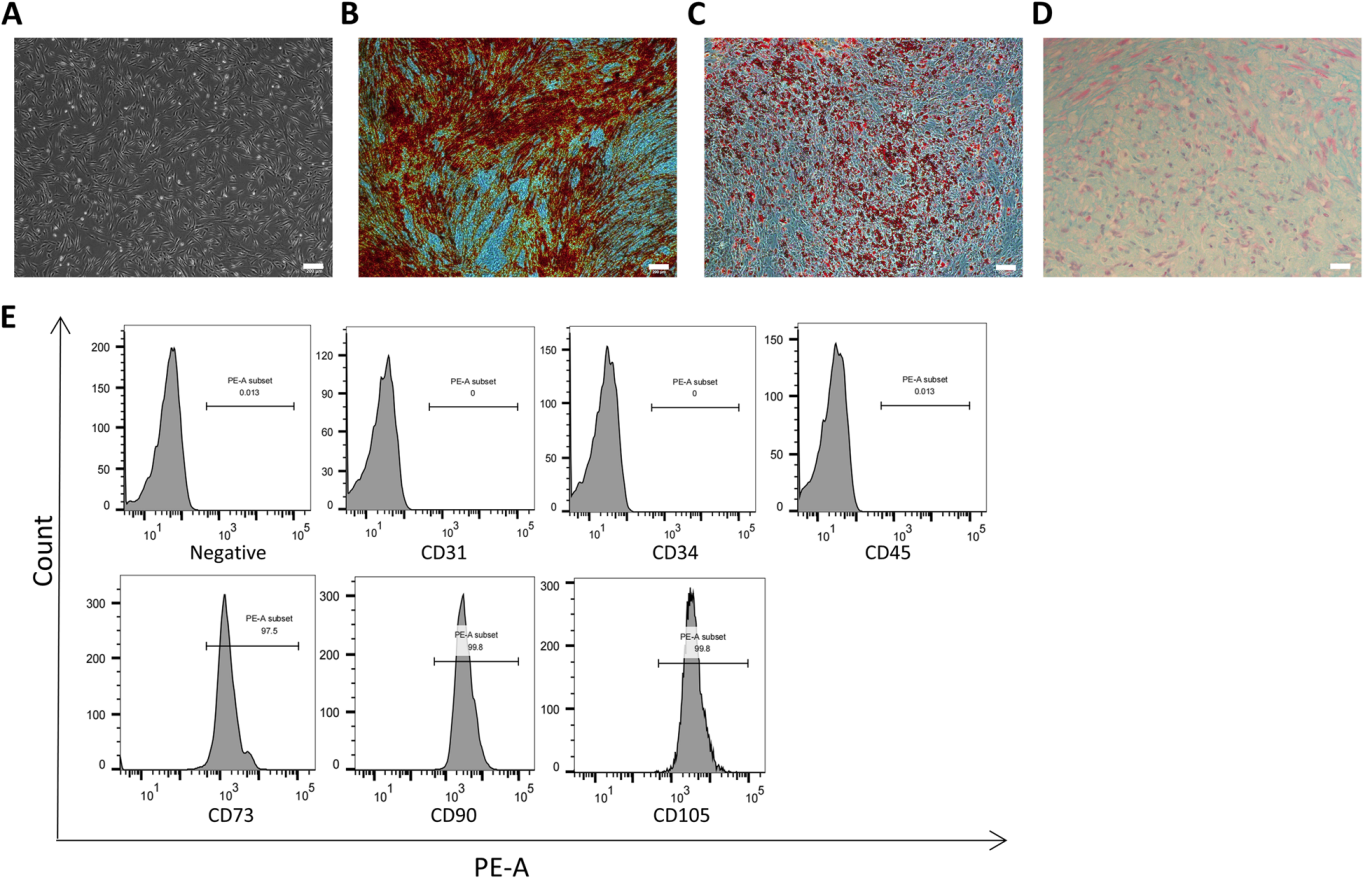
Characterization of ADSCs. **A**. Photomicrograph of adherent AD-MSCs with spindle shapes on a cell culture dish. Scale bar = 200 μm. **B**. Alizarin Red staining after osteogenic differentiation culture. Scale bar = 200 μm. **C**. Oil Red O staining after adipogenic differentiation culture. Scale bar = 200 μm. **D**. Alcian blue staining after chondrogenic differentiation culture. Scale bar = 50 μm. **E**. Flow cytometric characterization of ADSCs. CD105, CD90 and CD73 were positive, while CD34, CD31 and CD45 were negatively expressed.

Cultured primary and passaged ADSCs exhibited a spindle-shaped, fibroblast-like morphology, as shown in Fig 3A. We examined the multipotential differentiation capacity of ADSCs using adipogenic and osteogenic assays. The alizarin red stain showed evident orange calcium deposits and calcified nodules after being induced with an osteogenic medium for 21 days(Fig 3B). As is shown by Oil Red O staining, ADSCs developed an adipogenic phenotype after being induced with an adipogenic medium for 21 days(Fig 3C). We also cultured ADSCs with a chondrogenic medium for 3 weeks and stained them with Alcian blue, and the endoacid mucopolysaccharides were stained blue in cartilage globules (Fig 3D). The results demonstrated that the isolated ADSCs showed typical ADSCs characteristics.

ADSCs were positive for mesenchymal stem cells markers CD105(99.8%), CD90(99.8%), CD73(97.5%) and negative for hematopoietic stem cell markers CD45 (0.013%), CD31(0%), CD34(0%) as determined by flow cytometry(Fig 3E).

### Hypoxic pretreated ADSCs secrete more cytokines including VEGF by activating HIF1α

**Fig. 4:**
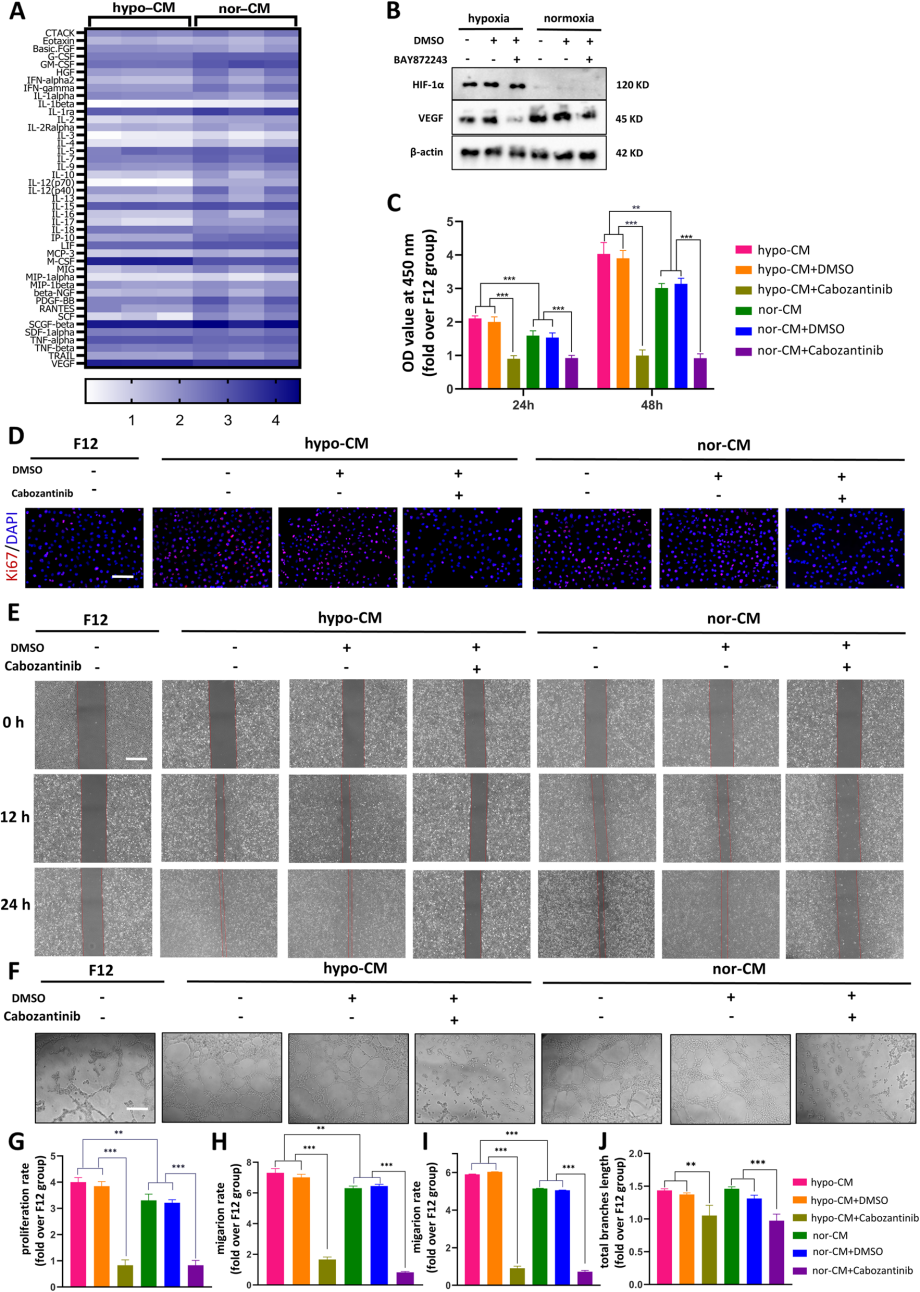
Hypoxic pretreated ADSCs secrete more cytokines and Hypo-CM promotes proliferation and migration of HUVECs. **A**. Heat map of the concentration of cytokines secreted by ADSCs under hypoxia and normoxia. **B**. Western blotting images of HIF-1α, VEGFA and β-actin from ADSCs treated with or without BAY87-2243 under hypoxia and normoxia. **C**. CCK-8 assay of HUVECs treated by F12, hypo-CM and nor-CM with or without cabozantinib at 24 h, and 48 h. **D**. Fluorescence images showing proliferation of HUVECs cultured by F12, hypo-CM and nor-CM with or without cabozantinib, respectively. (Red: Ki67, blue: DAPI). Scale bar = 100 μm. **E**. Representative images of the wound closure of HUVECs treated by F12, hypo-CM and nor-CM with or without cabozantinib at 0h, 12h, and 24h. Scale bar = 200 μm. **F**. Tube formation of HUVECs treated by F12, hypo-CM and nor-CM with or without cabozantinib at 10h. Scale bar = 50 μm. **G**. Statistical analysis of portion of Ki67 positive cells (fold over F12 group). **H** and **I**. Statistic alanalysis of migration area in scratch assay at 12h and 24h, respectively. (fold over F12 group). **J**. Statistical analysis of total branches length (fold over F12 group). The results are presented as mean ± SD (**P<0.01, ***P<0.001).

The ultrafiltration centrifugal tubes were used to collect cADSCs-CM. Luminex liquid suspension chip detection was used to detect the levels of 48 cytokines in hypo-CM and nor-CM. Among 48 cytokines evaluated, 5 cytokines were not detectably or showed extremely low expression (data not shown). 43 secreted cytokines were detected, and the data was converted into log10 form and displayed in the heat map. It can be seen in Fig 4A that a variety of growth factors and inflammatory factors were detected, and among them, VEGF was present at a high level Among them, VEGF, which is known to be one of the most important factors in promoting vascular regeneration, is present in high levels. Besides, the VEGF concentration of hypo-CM was much higher than that of nor-CM(8029.9±247.02 pg/ml VS 3757.3±261.8 pg/ml, *P<0.001*). Our results also showed that hypoxic pretreated ADSCs expressed high levels of G-CSF , GM-CSF, PDGF, and HGF, which have been proved to accelerate wound healing and proliferative capacity of skin cells such as fibroblasts. The concentration of these cytokines in hypo-CM was 201.8±3.5 pg/ml, 317.7±16.6 pg/ml, 165.5±13.5 pg/ml, and 49.2±7.04pg/ml, respectively, which is much lower than that of VEGF.

To investigate the reason why hypoxia treatment promotes the increase of VEGF, we added BAY87-2243(5 μM), a potent inhibitor of HIF1 that can only be dissolved in DMSO, to the F12 culture medium of ADSCs, and F12+DMSO was applied as a negative control. An inspection of Fig 4B reveals that HIF1α expression was only detectable in a hypoxic culture which is not surprising. Furthermore, the VEGF expression of hypoxic cultured ADSCs was significantly reduced by the addition of BAY87-2243, which was not significantly decreased in normoxic culture.

### Hypo-CM promotes proliferation and migration of HUVECs through VEGF/Akt/mTOR and MAPK signal pathway

To investigate whether VEGF plays a role in cADSCs-CM affecting HUVECs, cabozantinib at a concentration of 5 μM was used to inhibit the VEGFR2 receptor. We divided HUVECs into seven groups treated with F12, hypo-CM, hypo-CM+DMSO, hypo-CM+cabozantinib, nor-CM, nor-CM+DMSO, nor-CM+cabozantinib, respectively. The results of the CCK-8 assay demonstrated a significant increase in the optical density(OD) of HUVECs after being treated with hypo-CM and nor-CM, which implied an elevated cell count. Under conditions of consistent initial cell numbers, the hypo-CM group was significantly higher than the nor-CM group, both at 24 h and at 48 h. However, this proliferation promoting effect was completely inhibited after the addition of cabozantinib (Fig 4C). The proliferation of HUVECs was also shown by Ki67 immunofluorescence staining. The percentage of nuclei with red fluorescence which meant HUVECs in proliferating phase showed that hypo-CM and nor-CM improved the proliferation of HUVECs in several, and hypo-CM had the greatest proliferation promoting capacity. What stands out in the figure is that the proliferation-promoting effect of either hypo-CM or nor-CM disappeared after the addition of cabozantinib(Fig 4D, 4F).

The migration of HUVECs was evaluated by scratch assays. Each group was treated with F12, hypo-CM, hypo-CM+DMSO, hypo-CM+cabozantinib, nor-CM, nor-CM+DMSO, nor-CM+cabozantinib for 24 h after being scratched. No significant migration was found in F12, hypo-CM+cabozantinib, and nor-CM+cabozantinib groups at both time points. The wound closure ratio of hypo-CM treated HUVECs was remarkably higher than the other groups at 12 h and 24 h. Nor-CM-treated HUVECs also exhibited a high migration capacity, but the migration rate at both time points was inferior to that of the hypo group(Fig 4E, H, I).

With regard to the tubule formation experiment, not surprisingly, the results were similar to those of the proliferation and migration assay. After 10 h of treatment, hypo-CM- and nor-CM-stimulated HUVECs formed more capillary-like tubes earlier on Matrigel compared to F12 treatment. No capillary-like tube was found in hypo-CM+cabozantinib and nor-CM+cabozantinib groups(Fig 4F). Quantitative measurement that showed that the total branches length was presented in Fig 4J.

**Fig. 5:**
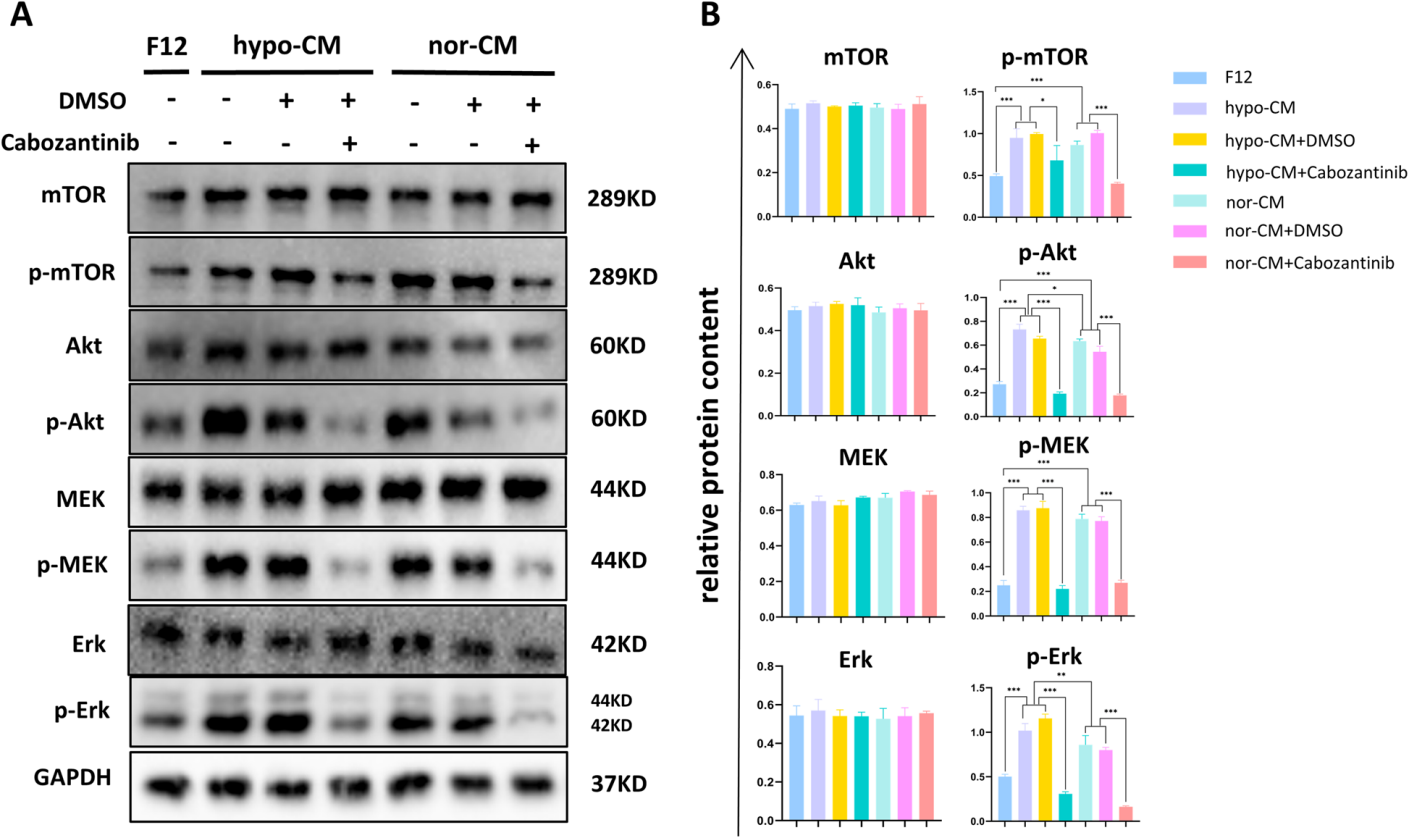
Detection of the Akt/mTOR and MAPK signaling pathway of HUVECs treated by F12, hypo-CM and nor-CM with or without cabozantinib. **A**. Western blotting images of AKT, mTOR, MEK, Erk, phospho-AKT, phospho-mTOR, phospho-MEK, phospho-Erk, and GAPDH. **B**. Statistical analysis of the protiens content relative to that of GAPDH. The results are presented as mean ± SD (*P<0.05, **P<0.01, ***P<0.001).

Since we found that hypo-CM and nor-CM promoted proliferation and migration of HUVECs in vitro, we further investigated its mechanisms. Since AKT/mTOR and MAPK signaling pathways play important roles in cell survival, proliferation and vascular development, we hypothesized that VEGF/AKT/mTOR and VEGF/MAPK signaling might be involved in the biological effects of hypo-CM and nor-CM on the HUVECs. As expected, western blot analysis showed that levels of phospho-AKT, phospho-mTOR, phospho-MEK, and phospho-Erk were increased in hypo-CM- and nor-CM-stimulated HUVECs compared to the F12 group. Then to elucidate if VEGF is necessary for the activation of AKT/ mTOR and MAPK signaling pathway, we treated HUVECs with cabozantinib. We found that inhibition of the VEGFR receptor by cabozantinib significantly inhibited the phosphorylation of AKT, mTOR, MEK, and Erk, suggesting that the activation of AKT/mTOR and MAPK signaling mediated by hypo-CM and nor-CM is associated with VEGF(Fig 5A, 5B).

### Characterization of GelMA+hypo-CM hydrogel

GelMA was synthesized using various concentrations of MA to create polymers with different degrees of methacrylation. As reported in already published research, the addition of methacrylate groups to the amine-containing side groups of gelatin can be used to make it light polymerizable into a hydrogel that is stable at 37 °C, and many key physical properties and molecular structures of GelMA with different degrees of methacrylation have been well characterized. In this study, we chose GelMA of different concentrations with an MA substitution degree of 60% to detect the mechanical properties.

**Fig. 6:**
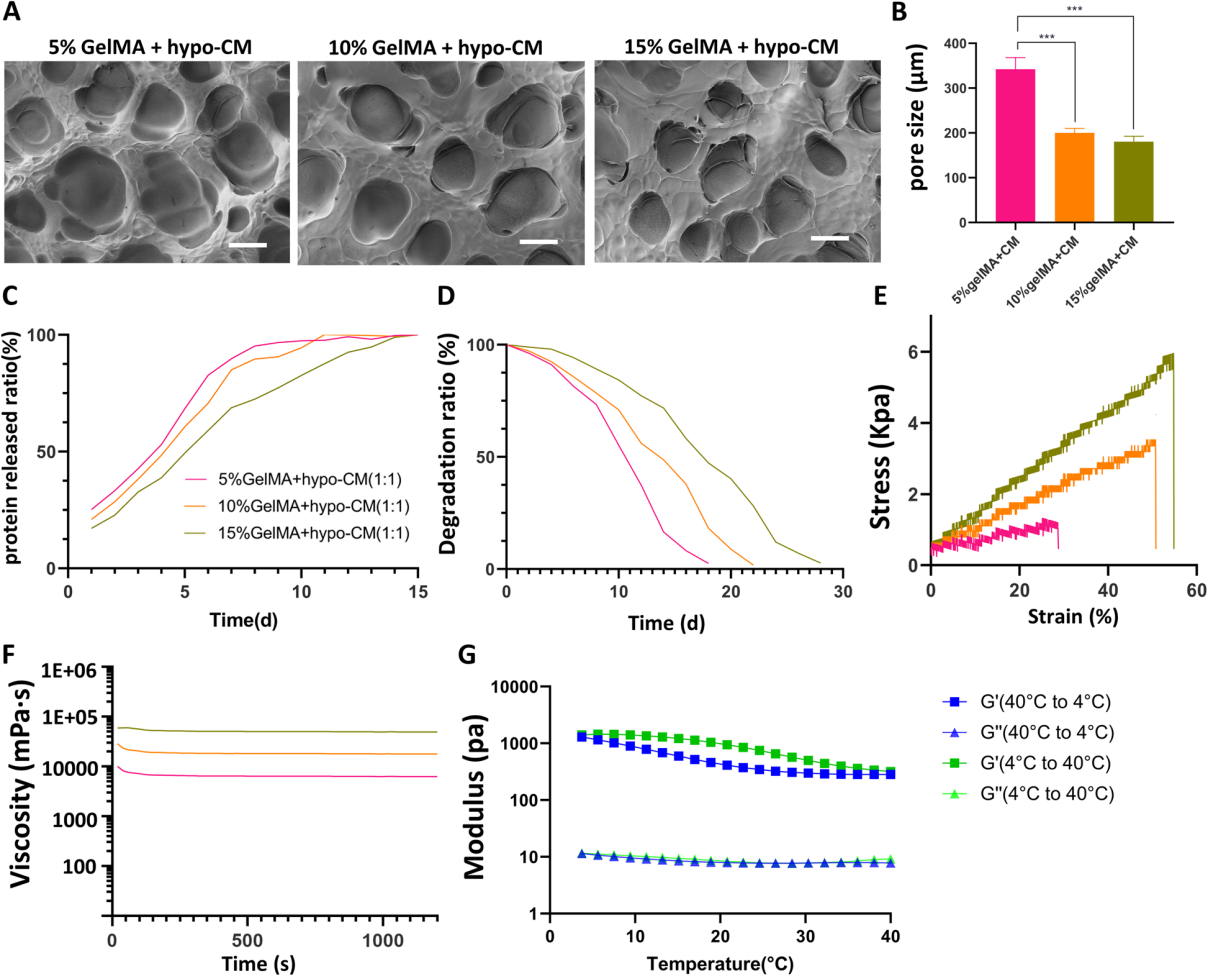
Characterization of GelMA+hypo-CM hydrogel. **A**. SEM images of different concentrations of GelMA loaded with hypo-CM. Scale bar = 100 μm. **B**. Mean pore size of different concentrations loaded with hypo-CM. **C**-**E**. Protein released ratio, degration ratio with time, stress-strain curves of GelMA+hypo-CM hydrogel. **F**. Viscosity curve with time of GelMA+hypo-CM hydrogel. **E**. Modulus change curve of 10% GelMA+hypo-CM hydrogel (temperature increase from 4 °C to 40 °C and decrease from 40 °C to 4 °C).

To find the appropriate wound dressing, we configured three concentrations(5%, 10%, 15%(w:v)) of GelMA, and hypo-CM was then mixed into GelMA with a ratio of 1:1(v:v). After photo-crosslinked and lyophilization, the porous structures were observed by SEM(Fig 6A). The surface morphology of the GelMA+hypoCM hydrogel scaffold exhibits a smooth, continuous, and interconnected porous three-dimensional network structure. The 5%, 10%, and 15% GelMA+ hypoCM hydrogel respectively had a pore size of 342.3 μm, 200.2 μm, and 180.4 μm with the difference being statistically significant(*P<0.001*)(Fig 6B).

In addition, we immersed the manufactured GelMA+ hypoCM hydrogel in PBS and assay the protein concentration released into PBS at predetermined time points using the BCA method to calculate the protein release profile. As shown in Fig 6C, all three concentrations of GelMA+ hypoCM hydrogel had higher release rates in the first few days, the burst release of 5% GelMA+ hypoCM hydrogel had a shorter duration of about 6 days, while the 10% and 15% GelMA+ hypoCM hydrogels released proteins in a more steady and sustained manner.

The degradation rate of the hydrogels was measured every 2 days during immersion in the culture medium (Fig 6D), where the 10% and 15% GelMA + hypoCM hydrogels’ degradation was much slower than the lower concentration group. The increased tensile strength with a higher concentration from 5% to 15% of GelMA + hypoCM hydrogel was revealed in Fig 6E. The elongation at break for 5%, 10%, and 15% GelMA + hypoCM hydrogel were 28.6%, 50.8%, and 54.5%, respectively.

Rheology is the science of material deformation and flow (continuous deformation), which occupies an important part in hydrogel research as a window to the internal structure of materials. Owing to the irregular shape of skin wounds, a hydrogel with self-healing ability is preferred. First, we performed a rotational shear test on the cross-linked GelMA + hypoCM hydrogels at a shear rate of 10/s to obtain a profile of viscosity versus time. As illustrated in Fig 6F, 15% GelMA + hypoCM hydrogels had the highest viscosity. Secondly, the rheological properties reflected by the changes in the storage modulus (G′) and loss modulus (G″) of the cross-linked 10% GelMA + hypoCM hydrogel were inspected. The temperature-dependent modulus change was studied to verify the constant self-assembly of the hydrogel after its formation. When the temperature increased from 4 °C to 40 °C, G′ decreased from 1407.7 Pa to 316.58 Pa, and G″ decreased from 11.62 Pa to 9.17 Pa under 1% strain and 5 Hz frequency. When the temperature reduced from 40 °C to 4 °C, G′ increased from 282.83 Pa to 1298 Pa, and G″ increased from 7.80 Pa to 11.40 Pa under 1% strain and 5 Hz frequency, indicating that the cross-linked 10% GelMA + hypoCM hydrogel remains solid at ambient temperatures of 4 - 40 °C.

### GelMA-hypoCM accelerates wound healing and angiogenesis in aged skin in vivo

**Fig. 7:**
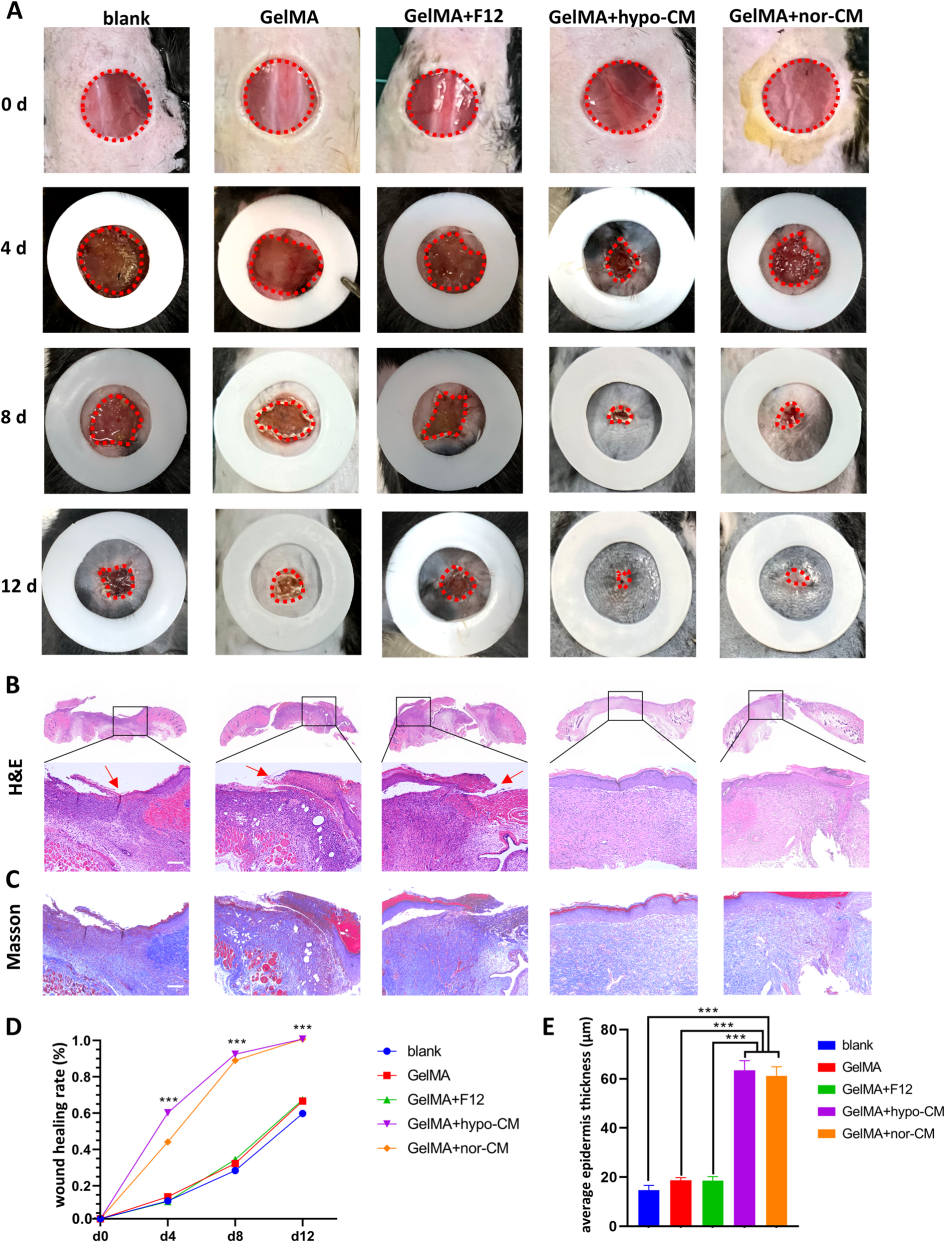
GelMA-hypoCM accelerated wound healing in aged skin in vivo. **A**. Gross view of wounds with diferent treatments on 4, 8, and 12 days post-wounding. **B** and **C**. H&E and Masson staining of wound site of differernt groups at day 12. Scale bar = 100 μm. (Red arrows indicate epithelial discontinuities). **D**. Statistical analysis of the wound healing rate. E. Statistical analysis of the average epidermis thickness. The results are presented as mean ± SD (***P<0.001).

A full-thickness skin wound model in C57BL/6J mice(20-month-old) was used to assess the effect of the GelMA + hypoCM hydrogel on wound healing in aged skins, and 10% GelMA was used in this study. Mice were randomly divided into 5 groups treated with a) blank; b) GelMA; c) GelMA+F12 group; d) GelMA+hypo-CM; e) GelMA+nor-CM. Digital photographs were obtained 0, 4, 8, and 14 days after surgery(Fig 7A). It is apparent from this figure that the wound healing process was accelerated most by GelMA+hypo-CM, it also showed improvement by GelMA+nor-CM. Especially on days 4 and 8, the GelMA+hypo-CM group demonstrated the fastest healing rate followed by the GelMA+nor-CM group. On day 12, the GelMA+hypo-CM – and GelMA+nor-CM-treated wounds healed up completely, but the other three groups still had a mostly unhealed wounds.

To further estimate the capability of the GelMA+hypo-CM hydrogel to promote healing, H&E, Masson, immunohistochemical staining of CD31, and blood perfusion evaluation were used to analyze the tissue sections of the wound bed on day 12. Wounds treated with GelMA+hypo-CM hydrogel or GelMA+nor-CM hydrogel have more granulation tissue to fill the defect and provide more blood supply, and a continuous epidermal layer was observed in both groups(Fig 7B). We also plotted a line graph of wound healing rates, from which it can be seen that the GelMA+hypo-CM and GelMA+nor-CM groups healed significantly faster than the other three groups at any predetermined time point. Moreover, the healing rate of the GelMA+hypo-CM group was faster than the GelMA+nor-CM group in the early stage of wound healing(Fig 7D). As shown in Fig. 7C, the wound bed of the GelMA+hypo-CM hydrogel or GelMA+nor-CM hydrogel treated mice presented thicker skin structures than other groups, and collagens in granulation tissue were also observed. We also measured the average epidermal thickness and, not surprisingly, the GelMA+hypo-CM and GelMA+nor-CM groups that had fully healed had thicker epidermis, but the difference between these two groups was not statistically significant.

**Fig. 8:**
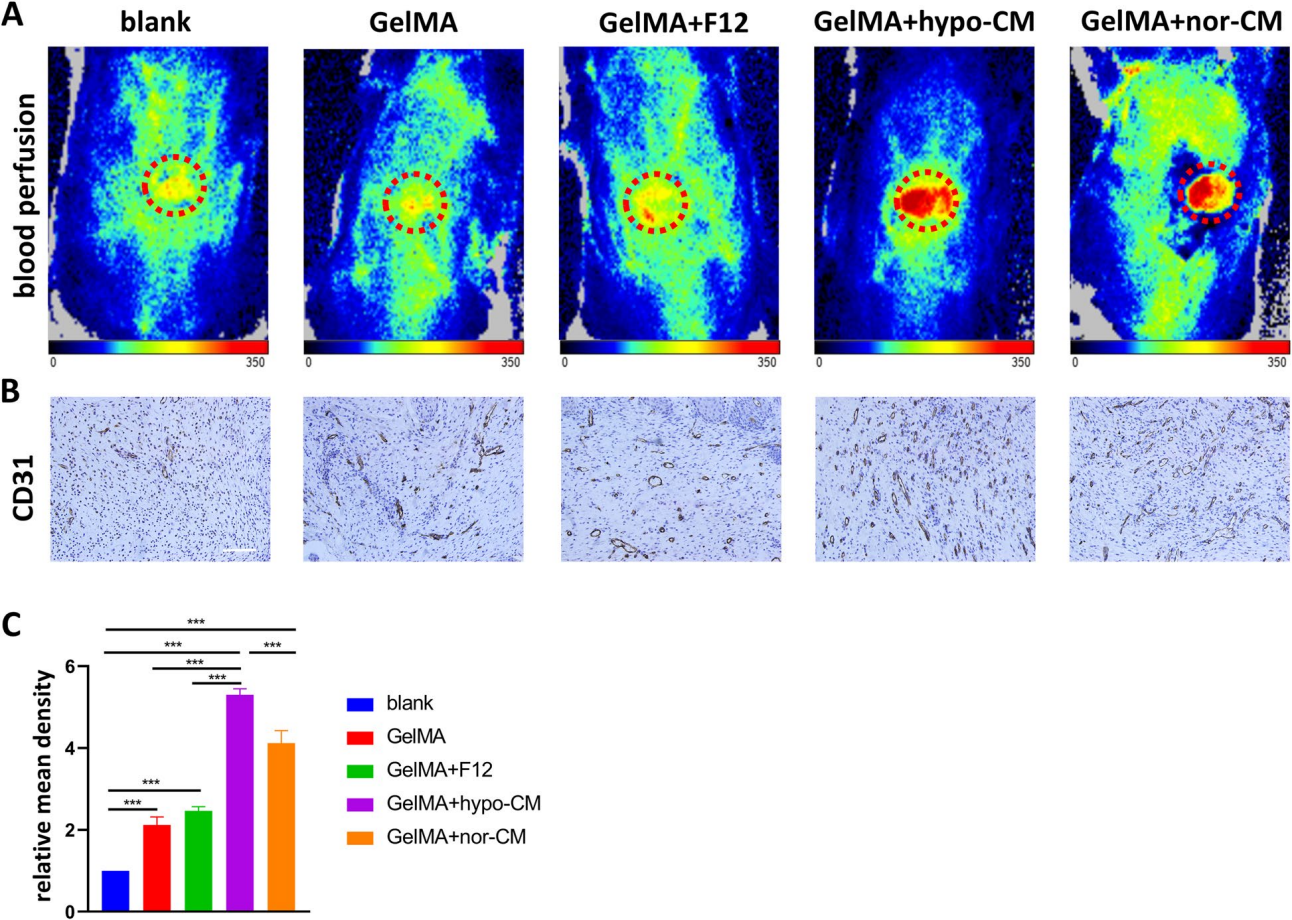
GelMA-hypoCM accelerates angiogenesis in aged skin in vivo. **A**. The blood perfusion at the wound areas of both groups were evaluated by a laser Doppler perfusion imaging system. **B**. Immunohistochemical staining of CD31 in regenerated skin tissue. Scale bar = 50 μm. **C**. Quantitative analysis of relative mean density of immunohistochemical staining of CD31 in regenerated skin tissue. The results are presented as mean ± SD (***P<0.001).

In vivo angiogenesis was assessed by the laser Doppler perfusion imager. Area and density of flux images of blood perfusion are represented in red. The GelMA+hypo-CM hydrogel or GelMA+nor-CM hydrogel treated wounds had elevated periwound levels compared to the other groups(Fig 8A). CD31 is the surface marker of vascular endothelial cells, which is a key marker of angiogenesis. From Fig 8B and 8C, we can note that an increased number of mature vessels with larger diameters was observed in the GelMA+hypo-CM hydrogel group compared to that of the GelMA+nor-CM hydrogel group. It also showed an increased number of microvessels in single GelMA or GelMA+F12 treated wounds compared to the blank group.

## Discussion

With the rapid increase in the number and proportion of the aging population, we have to face the burden of delayed-healing wounds on society and medicine, especially in developed countries. Cutaneous wound healing is a very complex process involving a large number of cell types, tissues, cytokines, and chemokines. It can be divided into three overlapping phases: hemostasis and inflammatory phase, proliferative phase, and remodeling phase [28, 29]. An optimal repair can only be reached with the precise regulation of various cells, cytokines, and tissues.

Older individuals exhibit alterations in cell function, including cell adhesion, proliferation, and migration, which affect the rate of wound healing [30, 31]. For example, previous studies showed that in the early stages of wound healing in aged skin, loss of neutrophil and macrophage function resulted in reduced secretion of many chemokines and slower cellular recruitment, which may lead to wound infection and subsequent chronic inflammation [32, 33]. Most importantly, the lack of neovascularization leads to insufficient oxygen content in the microenvironment, which may be attributed to insufficient endothelial cell recruitment or low VEGF levels. In a low oxygen environment, the energy produced by cellular glycolysis is not sufficient to support cellular activity, In addition, oxidative stress caused by the generation of reactive oxygen species(ROS) also degrades collagen [34]. Therefore, vascular regeneration is essential for wound healing in aged skin.

Mesenchymal stem cells(MSC) that are characterized by the ability to secrete large amounts of cytokines have shown outstanding performance in repairing chronic wounds. In this study, we successfully extracted and cultured human ADSCs. According to previous studies, MSCs should express several mesenchymal markers [35]. After identification, it was found that the markers of mesenchymal stem cells, such as CD105, CD90, and CD73, were highly expressed, while other cell markers, such as CD45, CD31, and CD34, were negative. In addition, ADSCs also have adipogenic and osteogenic differentiation abilities, which is consistent with previous studies. After that, we performed hypoxic and normoxic culture of ADSCs and collected ADSCs conditioned medium. We found that hypo-CM contained more cytokines and growth factors than nor-CM, such as VEGF. VGEF expression of hypoxic-pretreated ADSCs has significantly reduced after blocking HIF1 with BAY87-2243, predicting that the hypoxic environment activated the HIF1/VEGF pathway in ADSCs.

Furthermore, we evaluated the therapeutic and proangiogenic effects of hypo-CM in vitro, and the results confirmed that hypo-CM promoted the abilities of proliferation, migration, and tube formation of HUVECs better than nor-CM treatment. This finding is consistent with several previous research [36, 37]. To our knowledge, VEGFA binds to the VEGFR2 receptor on the cell membrane of HUVECs, thereby activating a series of signaling pathways related to cell survival, proliferation, and migration, such as Akt/mTOR and MAPK signal pathway [38]. For validation, we examined the content of proteins of relevant signaling pathways and the content of phosphorylated proteins using western blotting. Consistently, the Akt/mTOR and MAPK signal pathways of both hypo-CM-treated and nor-CM-treated HUVECs were activated. However, No significant proliferation, migration, or tube formation was detected after blocking the VGEFR2 receptor with the application of cabozantinib, which is consistent with phosphorylated proteins. Our findings would seem to suggest that hypo-CM and nor-CM could promote proliferation, migration, and tube formation through VEGF/ Akt/mTOR and MAPK signal pathways.

Even though there have been many studies of stem cell conditioned medium or extracellular vesicles for the treatment of wounds, this technology still has limits. A challenge that cannot be ignored is the difficulty of retaining conditioned medium in vivo, and the current solution is multiple injections at the edge of the wound or at the substrate, which undoubtedly results in waste. Therefore, it is urgent to find an injectable biomaterial that can release its contents continuously. GelMA is a photopolymerizable hydrogel comprised of modified natural extracellular matrix components, making it a potential material for tissue repair. GelMA crosslinks rapidly after a few seconds of UV irradiation and remains crosslinked at 37°C. It also resists degradation by proteolytic enzymes such as gelatinase and collagenases. As confirmed by previous literature, the mechanical properties and pore sizes in GelMA hydrogel can be tuned by changing the degree of methacryloyl substitution [39, 40]. GelMA hydrogel shows excellent biocompatibility and biosafety. As far as the composition is concerned, GelMA is synthesized by the direct reaction of gelatin with methacrylic anhydride (MA) in phosphate buffer at 50 °C. This reaction introduces methacryloyl substitution groups on the reactive amine and hydroxyl groups of the amino acid residues. It is worth mentioning that the chemical modification of gelatin by MA generally only involves less than 5% of the amino acid residues in molar ratio, which implies that most of the functional amino acid motifs [41, 42]. Second, a low concentration of photoinitiator is added in order to make it curable by visible light. Lithiumphenyl-2,4,6-trimethylbenzoyl phosphinate (LAP) are the most commonly used ones owed to their excellent biocompatibility and minimal immunogenicity [43], which allows GelMA to be crosslinked by blue light at a wavelength of 405 nm. In terms of application, gelMA has been a hot research topic in recent years, and has been applied as a drug carrier or scaffold material for the repair of various tissues and organs(Such as bone [44], cartilage [45], skin [46], heart [47], nerve [48] and blood vessel tissue [49]). This certainly illustrates the biosafety of GelMA.

Considering that our mixing of GelMA and hypo-CM in the ratio of 1:1 would dilute the GelMA and thus reduce the mechanical strength, we chose the gelma with 60% degree of methacryloyl substitution. The mechanical properties, rheology, porosity, and cytotoxicity of different concentrations of GelMA have been characterized in much previous literature [20, 22]. We mixed different concentrations of GelMA with hypo-CM in a 1:1(v:v) ratio. After a comprehensive evaluation of morphology, protein release efficiency, degradation rate, mechanical properties, and rheology properties, we found that the properties of GelMA were not altered. Finally, we chose 10% GelMA loaded with hypo-CM of which viscosity and duration of protein release are suitable to prepare a dressing.

To further confirmed the effects of GelMA+ hypo-CM hydrogel on wound healing in aged skin, full-thickness skin wounds were created on the backs of 20-month-old mice. After surgery, GelMA, GelMA+F12, GelMA+hypo-CM, and GelMA+nor-CM were applied to the wound surface respectively, and a blank group without treatment was used as a blank control. It was illustrated in Fig 7 that wounds treated with GelMA+hypo-CM had the fastest healing speed followed by that treated with GelMA+nor-CM compared to GelMA and GelMA+F12 groups. Re-epithelialization and collagen deposition demonstrated by HE and Masson staining corroborated the excellent results of GelMA+hypo-CM hydrogel, while discontinuous epithelia were observed in the other three groups. Although complete healing was achieved in both the GelMA+hypo-CM and GelMA+nor-CM groups by day 12 after treatment, the GelMA+hypoCM group had a faster healing rate in the early stages of wound healing. The reason may be the higher VEGF content in GelMA+hypoCM. The GelMA+norCM group also had a good treatment effect, which was evident. A number of researchers have treated refractory wounds with nor-CM(which was called ADSCs condition midium in other studies) and achieved good results [36]. The more surprising result was that the wounds treated with GelMA or GelMA+F12 hydrogel also demonstrated more rapid wound healing compared to the blank group. A possible explanation for this might be that GelMA, as a bioactive material, could protect would site from contamination outside and provide a better microenvironment for cells involved in wound healing.

The neovascularization and blood perfusion in the repaired area was increased by GelMA+hypo-CM hydrogel. Immunohistochemical staining showed more neovascular after being treated with GelMA+hypo-CM hydrogel. This implies that GelMA+hypo-CM hydrogel has a stronger pro-angiogenic capacity. The new capillaries provide more oxygen and nutrients to the cells at the wound site, which may also account for the faster early healing in the GelMA+hypo-CM group. In summary, the GelMA+hypo-CM hydrogel has better efficacy and application prospects. Our results are consistent with the findings of a great deal of previous work by Wang et al [25]. They reported that GelMA loaded with engineered extracellular vesicles could accelerate diabetic wound healing by promoting angiogenesis. It confirms the great potential of GelMA in the treatment of chronic wounds.

Given that GelMA is biocompatible, biodegradable, non-cytotoxic and non-immunogenic ,it has been used in many aspects of tissue engineering in recent years, especially for vascular reconstruction, which has been reported by many investigators. A 3D vascular network was constructed by applying GelMA by Nikkhah et al [50]. Lin et al [51] constructed GelMA loaded with human mesenchymal stem cells and human endothelial colony-forming cells and found that it could promote angiogenesis in vivo and in vitro. It has also been demonstrated that GelMA can provide the environment for HUVEC to form monolayers of cells and provide conditions for angiogenesis [52].

We were aware that our research may have a limitation that only one type of cell was considered in this study. Considering the complexity of wound healing, more cells such as fibroblasts, epidermal stem cells, etc. should be tested in the future.

## Conclusion

In this study, we found that GelMA loaded with hypo-CM or nor-CM could accelerate wound healing in aged skin by promoting angiogenesis, and GelMA loaded with hypo-CM had more remarkable effects possibly due to the higher content of VEGF. We supposed that the mechanism may be that VEGF in hypo-CM activates the Akt/mTOR and MAPK signaling pathway and promotes biological activities such as proliferation and migration of endothelial cells, leading to more adequate blood transport at the impaired site and ultimately promoting wound healing.

## Supplementary Information

**Additional file 1**. Full blots of western blot images.

## Data Availability

All data generated or analyzed during this study are included in this published article.
