# Supplementary figures and images for: Gelatin methacryloyl (GelMA) loaded with concentrated hypoxic pretreated adipose-derived mesenchymal stem cells(ADSCs) conditioned medium promotes wound healing and vascular regeneration in aged skin
Source: Biomater Res. 2023 Feb 13;27:11. doi: 10.1186/s40824-023-00352-3 (PMC9926638; doi:10.1186/s40824-023-00352-3)

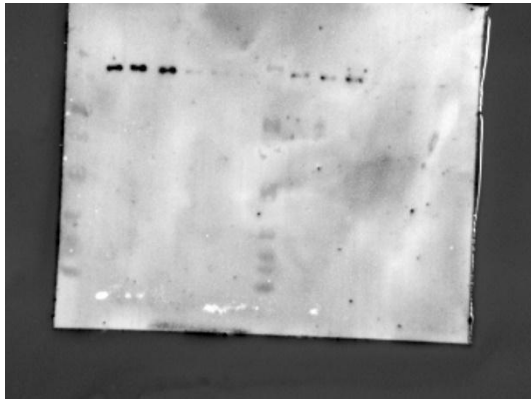

HIF1 $\alpha$

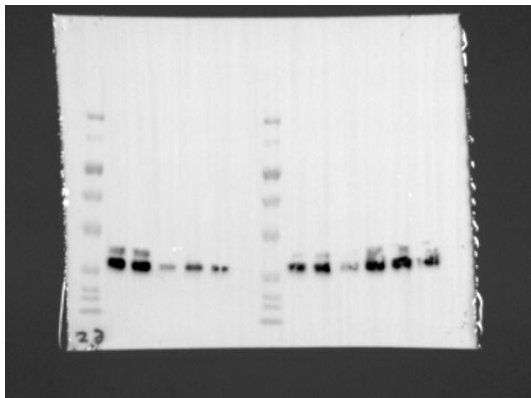

VEGF

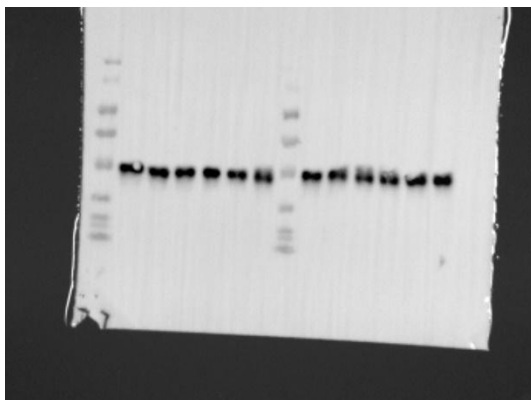

$\beta$ -actin

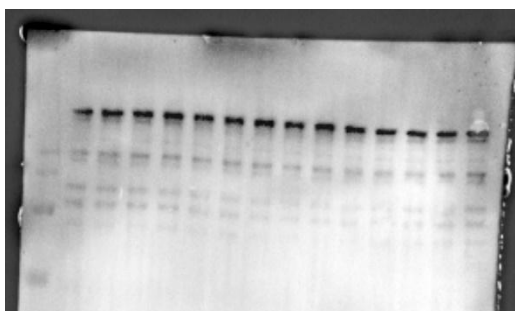

mTOR

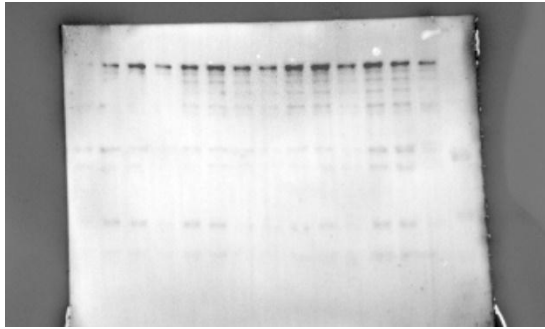

p-mTOR

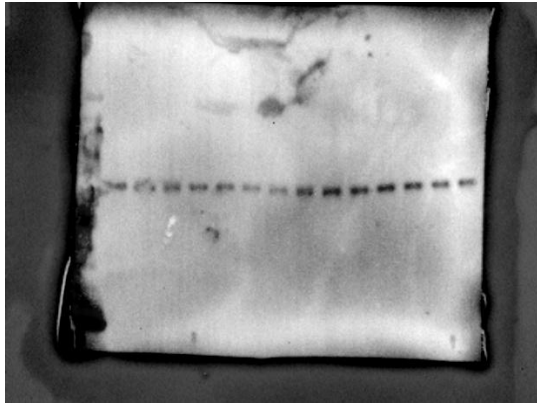

Akt

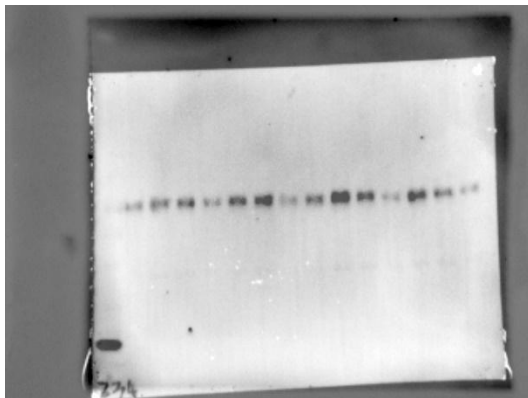

p-Akt

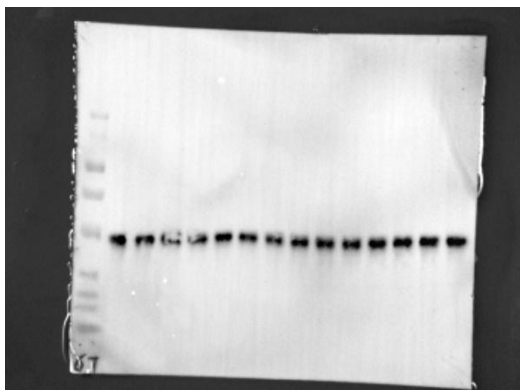

MEK

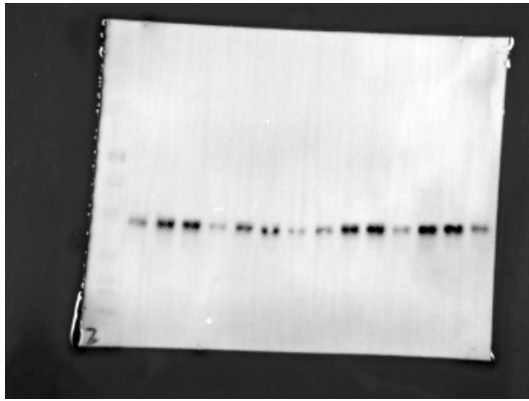

p-MEK

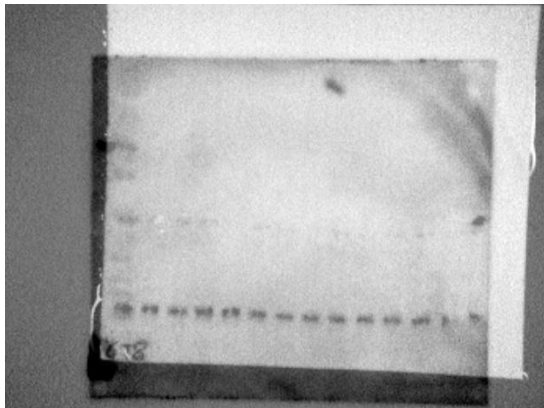

Erk

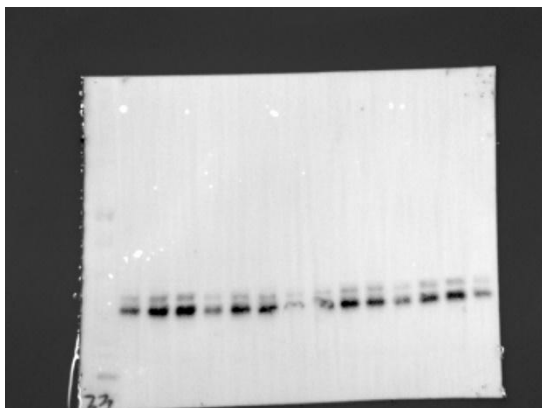

p-ERK

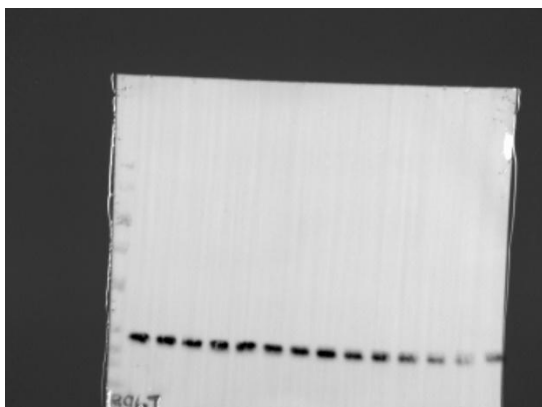

Gapdh

Supplement: Supplementary file 1 — Additional file 1. Full blots of western blot images. [file 40824_2023_352_MOESM1_ESM.pdf]
